# A Low-Cost Resource Re-Allocation Scheme for Increasing the Number of Guaranteed Services in Resource-Limited Vehicular Networks

**DOI:** 10.3390/s18113846

**Published:** 2018-11-09

**Authors:** Yun Meng, Yuan Dong, Chunling Wu, Xinyi Liu

**Affiliations:** 1 The Faculty of the Institute of Electrical and Control Engineering, Chang’an University, Xi’an 710064, China; dongyuan566@126.com (Y.D.); wuchl@chd.edu.cn (C.W.); 2 The Faculty of the Institute of Information Engineering, Chang’an University, Xi’an 710064, China; liuxinyi@chd.edu.cn

**Keywords:** resource-limited, re-allocation, service guaranteed

## Abstract

Vehicular networks are becoming increasingly dense due to expanding wireless services and platooning has been regarded as a promising technology to improve road capacity and on-road safety. Constrained by limited resources, not all communication links in platoons can be allocated to the resources without suffering interference. To guarantee the quality of service, it is required to determine the set of served services at which the scale of demand exceeds the capability of the network. To increase the number of guaranteed services, the resource allocation has to be adjusted to adapt to the dynamic environment of the vehicular network. However, resource re-allocation results in additional costs, including signal overhead and latency. To increase the number of guaranteed services at a low-cost in a resource-limited vehicular network, we propose a time dynamic optimization method that constrains the network re-allocation rate. To decrease the computational complexity, the time dynamic optimization problem is converted into a deterministic optimization problem using the Lyapunov optimization theory. The simulation indicates that the analytical results do approximate the reality, and that the proposed scheme results in a higher number of guaranteed services as compared to the results of a similar algorithm.

## 1. Introduction

### 1.1. Motivation

The demand for ubiquitous mobile services in an intelligent transportation system (ITS) is ever increasing to improve road safety and transport management [1]. Vehicle-to-vehicle (V2V) and vehicle-to-infrastructure (V2I) communications have attracted significant attention in vehicular networks [2,3]. V2V allows vehicle users to communicate directly within a group. This not only decreases the path loss by reducing the distance, but is also appropriate for many vehicular applications, including hazard warnings, path planning, vision sharing, and platooning [4,5]. V2I enables vehicle users to access roadside units (RSUs) for downloading or uploading data, which improves the stability of communication for vehicular users (VUs) with high mobility. Therefore, the efficiency and reliability of a vehicular network can be improved significantly by the cooperation between V2V and V2I networks [6].

Interference represents a problem in vehicular networks due to the increase in the density of VUs [7]. Resource allocation is a promising approach for interference management [8]. However, as a result of the rapidly growing demand for services, radio resources are scarce [9]. Thus, not all communication links can be allocated to a resource without suffering interference in dense networks. To guarantee the quality of services, the set of served services at which the scale of demand exceeds the service capability of the network has to be determined. To increase the number of guaranteed services, the resource allocation has to be adjusted to adapt to the dynamic environment of the vehicular network. However, frequent and uncontrolled resource re-allocation may bring about a large number of control signaling exchanges. Our goal in this paper is to devise a low-cost resource re-allocation scheme that reduces the re-allocation rate and increases the number of guaranteed services.

### 1.2. Related Work

Resource allocation in resource-limited vehicular networks with a selected set of served services is an important topic and has been well studied [10,11,12,13,14,15,16,17,18,19]. The optimization objective in References [10,11,12,13] was the maximization of the network capacity. A resource allocation scheme with flexible node grouping was proposed in Reference [10] to utilize resources in a more efficient manner. To meet different requirements for different vehicular links, the authors of References [11,12] maximized the ergodic capacity of V2I links while ensuring the reliability for each V2V link with a robust resource allocation. Considering the effect of mobility on the interference relationship, an iterative algorithm based on a time interval-dependent interference graph was proposed in Reference [13]. In References [14,15], the minimization of delay was investigated. The authors of Reference [14] proposed a radio resource scheduling scheme based on stochastic learning. The scheme in Reference [15] selected the optimal receiver to establish device-to-device links and assigned the proper channels by solving a maximum weighted independent set problem. The network utility was optimized in References [16,17,18]. The utility function in Reference [16] considered several key factors related to video quality, including the location, velocity, intensity, and smoothness requirements. The cloud resource allocation proposed in Reference [17] was modeled as a non-cooperative cloud resource allocation game based on the Gauss–Seidel iteration method, in which the utility reflects the performance of the user including the rate and delay. In Reference [18], the access mode selection and resource allocation scheme were formulated as a user aggregate utility maximization problem, taking into consideration the transmission delay, various network topologies, and power reduction. In Reference [19], the users’ secrecy rate was optimized by a joint power and subcarrier allocation based on a maximum-minimum fairness criterion. In the above mentioned studies, the resource re-allocation has not been considered. However, due to the high mobility of vehicles, the topology of vehicular networks is highly dynamic [20]. Without the latest environmental information, the resource allocation based on outdated information may not provide an optimal performance. Therefore, to maintain an outstanding performance of resource allocation, it is necessary to execute resource re-allocation frequently. Nevertheless, frequent resource re-allocation may result in a large number of the control signaling exchanges, which leads to a large overhead and degraded latency performance.

Several studies have investigated the problem of reducing resource re-allocation in vehicular networks [21,22,23]. The resource allocation scheme proposed in Reference [21] was based on calculating the similarity between interference state maps; re-allocation was started if the difference between the maps in two consecutive time slots exceeded a given threshold. Although the proposed scheme reduced the re-allocation in a network, the guarantee of the quality of service was not considered. Another study [22] minimized the re-allocation rate in the V2V network that underlays the cellular network. The resource allocation scheme in Reference [23] maximized the average service rate of all users with the restriction of the re-allocation rate in the virtual V2V network. The original optimization problems in References [22,23] were transformed to deterministic optimization problems using the Lyapunov optimization. However, in these two studies, it was assumed that the resources were sufficient and thus the data queues of all the services could kept stable in the network; this assumption is not applicable to future vehicular networks, in which resources are insufficient compared with the rapidly growing demand for services.

Platooning has been regarded as a promising technology to improve road capacity and on-road safety in ITSs [24,25,26,27]. References [24,25] investigated the performance including latency, reliability, and stability in the platooning. A hybrid security protocol was proposed for platoon communication aiming at ensuring platoon stability in Reference [26]. A resource allocation scheme in Reference [27] was proposed to reduce the transmission delay, the number of transmission hops and the transmission powers for a multi-platooning in a cellular network, although the guarantee for services and the re-allocation were not considered in this scheme. As a promising mathematical tool, stochastic geometry is beneficial to analyze the average performance of vehicular networks, since it can be evaluated quickly and it can be used to explore the performance optimization of the most relevant metrics [28,29,30]. One study [28] proposed a stochastic geometry approach to model the temporal and spatial behavior of vehicular networks. Based on an interference analysis using stochastic geometry, a Geo-Location access scheme was presented in Reference [29]. Another study [30] analyzed the capture probability, average throughput, and mean density using stochastic geometry. Therefore, to analyze the interference in a network consisting of platoons of vehicles, stochastic geometry is used in this paper.

### 1.3. Contributions

In order to provide efficient services in a resource-limited vehicular network, we employ stochastic geometry to analyze the interference in the platooning of vehicles. We then present a resource allocation optimization method that reduces the re-allocation rate and increases the number of guaranteed services. The key contributions of this study are the following:

1. The co-layer and cross-layer interferences in the vehicular network are quantitatively analyzed using stochastic geometry.
2. To guarantee the services in the dynamic network, the definition of the guaranteed service is given based on the changes in the data queue.
3. To increase the number of guaranteed services at a low cost in a resource-limited vehicular network, we propose a time dynamic optimization method that constrains the network re-allocation rate.
4. To decrease the computational complexity, we convert the proposed time dynamic optimization problem into a deterministic optimization problem using the Lyapunov optimization theory to determine the set of served services based on the dynamic changes in the network at each slot.

### 1.4. Organization

The remainder of this paper is organized as follows. In Section 2, we present the system model and formulate the channel allocation problem under the constraint of the re-allocation rate. In Section 3, we propose the solution framework based on the Lyapunov optimization. In Section 4, we present and discuss the simulation results. Finally, Section 5 concludes the paper.

## 2. System Model and Dynamic Optimization Problem

### 2.1. Network Model

**Figure 1:**
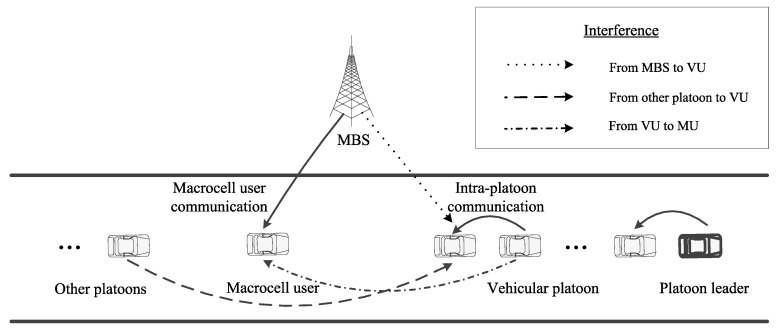
Scenario: The cellular vehicle-to-everything (C-V2X) network including platoons consists of vehicle users (VUs) and the underlying macrocell user communications.

As shown in Figure 1, this study considers a cellular vehicle-to-everything (C-V2X) scenario consisting of a macrocell and several platoons of vehicles on a freeway [31], where the V2I communication coexists with the V2V communication. The platoons share the channels with the macrocell users (MUs), i.e., the vehicle network underlies the cellular communication. It is assumed that the vehicles of a platoon have the same mobility patterns. This network uses Mode 4 (autonomously scheduled) for V2V communications to execute the resource allocation, i.e., the VUs determine the resource allocation themselves. Each platoon has a leader that gathers channel state information and allocates the channels to the platoon members. In the platoon, it is assumed that each vehicle communicates with the following one and sends the channel state information directly to the leader. There is no signal transmission among different platoons. Therefore, the channel allocations are independent in the different platoons. To mitigate the interference among the members of a platoon, the leader allocates orthogonal channels to the members. In the macrocell, the users are distributed along the road in the coverage of the base station; the MUs in the same macrocell base station (MBS) are assigned orthogonal channels. The downlink of the network is considered.

The platoons are modeled as a one-dimensional Poisson point process (1D PPP) Φ with a density λ (platoons/m), which is considered suitable for a freeway scenario [22,23]. The set of vehicles in a platoon is denoted as N, where the cardinality of N is denoted as *N*. The number of MUs and platoons are denoted as $N^{M}$ and $N^{P}$, respectively. White noise is neglected at the receivers. Let $K=\left\{1,2,\ldots ,K\right\}$ be the set of orthogonal channels. The threshold of the signal-to-interference ratio (SIR) is denoted as θ; that is, the packet transmission is successful only if the SIR is larger than θ. Let $P^{M}$ and $P^{V}$ denote the transmit power of the MBS and the VU, respectively, and let α denote the factor of the path loss. In this study, the small-scale fading of each channel denoted as *g* is distributed independently and identically and has an exponential distribution with a unitary mean [22,23], i.e., g∼exp(1).

### 2.2. Signal Model

For member *n* in the platoon, the interference from the MBS in channel *k* is:

(1) $$I_{nk}^{MV}=\left\{\begin{array}{c} P^{M}g_{k}^{MV}{\left(d^{MV}\right)}^{-\alpha },\mathrm{when}\;\mathrm{the}\;\mathrm{channel}\;k\;\mathrm{is}\;\mathrm{occupied}\;\mathrm{by}\;\mathrm{MUs},\\ 0,\;\mathrm{otherwise}, \end{array}\right.$$

where $g_{k}^{MV}$ and $d^{MV}$ represent the small-scale fading and the distance between the MBS and member *n* in the platoon. Compared to the distance between the MBS and the victim VU, the distance among the VUs is comparatively small. Thus, it is noted that the platoon is regarded as one point for the macrocell base station. Similarly, the platoon is regarded as one point for other platoons. The probability of a channel being occupied by an MU is denoted as $p^{M}$.

For member *n* in the platoon, the aggregated interference from the other platoons in channel *k* is:

(2) $$I_{nk}^{VV}=\sum_{q\in \Phi }P^{V}g_{qk}^{VV}{\left(d_{q}^{VV}\right)}^{-\alpha },$$

where $g_{qk}^{VV}$ and $d_{q}^{VV}$ represent the small-scale fading and the distance between the interferer and member *n* in the platoon.

For member *n* in the platoon, the total aggregated interference from the MBS and the other platoons in channel *k* is represented as:

(3) $$\begin{array}{cc} I_{nk}^{V}= & \;I_{nk}^{MV}+I_{nk}^{VV}\\ = & \;I_{nk}^{MV}+\sum_{q\in \Phi }P^{V}g_{qk}^{VV}{\left(d_{q}^{VV}\right)}^{-\alpha }. \end{array}$$

Therefore, the SIR of member *n* in the platoon in channel *k*, which is denoted as $SIR_{nk}^{V}$, can be derived as:

(4) $$\begin{array}{cc} SIR_{nk}^{V}= & \;\frac{S_{nk}^{V}}{I_{nk}^{V}}\\ = & \;\frac{P^{V}g_{nk}^{V}{\left(d^{V}\right)}^{-\alpha }}{I_{nk}^{MV}+\sum_{q\in \Phi }P^{V}g_{qk}^{VV}{\left(d_{q}^{VV}\right)}^{-\alpha }}, \end{array}$$

where $g_{nk}^{V}$ and $d^{V}$ represent the small-scale fading and the distance between the corresponding transmitter and member *n* in the platoon.

### 2.3. Availability Probability Calculated by Stochastic Geometry

To control the interference in the vehicular network, the access control criterion is proposed in this section. Channel *k* is available to member *n* in the platoon when the following two conditions are satisfied simultaneously.

Condition 1: Channel *k* is not occupied by the MU or the usage of channel *k* for member *n* in the platoon cannot cause strong interference with the MUs, which is guaranteed by the SIR threshold θ. This condition is written as:

(5) $$\left\{\begin{array}{c} \mathrm{the}\;\mathrm{channel}\;k\;\mathrm{is}\;\mathrm{not}\;\mathrm{occupied}\;\mathrm{by}\;\mathrm{MUs},\;\mathrm{or}\;\\ \frac{P^{M}g_{k}^{M}{\left(d_{k}^{M}\right)}^{-\alpha }}{P^{V}g_{k}^{VM}{\left(d^{VM}\right)}^{-\alpha }}\geq \theta ,\;\mathrm{when}\;\mathrm{the}\;\mathrm{channel}\;k\;\mathrm{is}\;\mathrm{occupied}\;\mathrm{by}\;\mathrm{MUs}, \end{array}\right.$$

where $g_{k}^{M}$ and $d_{k}^{M}$ represent the small-scale fading and the distance between the MBS and the corresponding MU that is active in channel *k*. The symbols $g_{k}^{VM}$ and $d_{k}^{VM}$ represent the small-scale fading and the distance between the VU interferer and the MU that is active in channel *k*.

Condition 2: Channel *k* is not occupied by the MU or the ratio of the interference from the MBS to the interference from the other platoons is smaller than a threshold β:

(6) $$\left\{\begin{array}{c} \mathrm{the}\;\mathrm{channel}\;k\;\mathrm{is}\;\mathrm{not}\;\mathrm{occupied}\;\mathrm{by}\;\mathrm{MUs},\;\mathrm{or}\;\\ \frac{I_{nk}^{MV}}{I_{nk}^{VV}}\leq \beta ,\;\mathrm{when}\;\mathrm{the}\;\mathrm{channel}\;k\;\mathrm{is}\;\mathrm{occupied}\;\mathrm{by}\;\mathrm{MUs}. \end{array}\right.$$

Condition 1 restricts the interference from VU to MU and Condition 2 restricts the interference from MBS to VU. Then, each platoon leader determines the set of available channels according to the above two conditions. Conditions 1 and 2 require information including the locations of MUs and MBS and the channel occupation of all MUs. Therefore, at the beginning of each time slot, the MBS broadcasts information on the geographical location and channel occupation of all MUs to each platoon leader. To decrease the overhead, this transmission of the control information is one-way; that is, only the MBS broadcasts the control information and there is no feedback. After receiving the control information, the platoon leader makes a decision regarding the set of available channels.

The MBS allocates channels to the MUs by self-determination. Let $b_{k}$ denote the channel allocation for the MUs in channel *k*, which is defined as:

(7) $$b_{k}=\left\{\begin{array}{c} 1,\;\mathrm{if}\;\mathrm{channel}\;k\;\mathrm{is}\;\mathrm{allocated}\;\mathrm{to}\;\mathrm{MU},\;\\ 0,\;\mathrm{otherwise}. \end{array}\right.$$

Therefore, according to Equations (5)–(7), the availability probability denoted as $\rho_{nk}$ can be derived as:

(8) $$\begin{array}{cc} \rho_{nk}= & \;1-b_{k}+b_{k}P\left(\frac{P^{M}g_{k}^{M}{\left(d_{k}^{M}\right)}^{-\alpha }}{P^{V}g_{k}^{VM}{\left(d_{k}^{VM}\right)}^{-\alpha }}\geq \theta \right)P\left(\frac{I_{nk}^{MV}}{I_{nk}^{VV}}\leq \beta \right)\\ = & \;\rho_{nk}^{1}\rho_{nk}^{2}, \end{array}$$

where $\rho_{nk}^{1}=P\left(\frac{P^{M}g_{k}^{M}{\left(d_{k}^{M}\right)}^{-\alpha }}{P^{V}g_{k}^{VM}{\left(d_{k}^{VM}\right)}^{-\alpha }}\geq \theta \right)$ and $\rho_{nk}^{2}=P\left(\frac{I_{nk}^{MV}}{I_{nk}^{VV}}\leq \beta \right)$.

Let the symbol $z=\frac{g_{k}^{VM}}{g_{k}^{M}}$ . As the small-scale fading of $g_{k}^{VM}$ and $g_{k}^{M}$ are independent and g∼exp(1), then, according to the distribution of random variables function, the probability density function of *z* can be derived as follows:

(9) $$\begin{array}{cc} f\left(z\right)= & \;\int_{0}^{+\infty }g_{k}^{M}\exp\left(-g_{k}^{M}z\right)\exp\left(-g_{k}^{M}\right)dg_{k}^{M}\\ = & \;\frac{1}{{\left(1+z\right)}^{2}}. \end{array}$$

The distribution function of *z* is denoted as $F_{z}\left(z_{0}\right)=\int_{-\infty }^{z_{0}}f\left(z\right)dz$. Then, $\rho_{nk}^{1}$ can be obtained as:

(10) $$\begin{array}{cc} \rho_{nk}^{1}= & \;P\left(\frac{g_{k}^{VM}}{g_{k}^{M}}\leq \frac{P^{M}{\left(d_{k}^{M}\right)}^{-\alpha }}{\theta P^{V}{\left(d_{k}^{VM}\right)}^{-\alpha }}\right)\\ = & \;F_{z}\left(\frac{P^{M}{\left(d_{k}^{M}\right)}^{-\alpha }}{\theta P^{V}{\left(d_{k}^{VM}\right)}^{-\alpha }}\right)\\ = & \;\frac{P^{M}{\left(d_{k}^{M}\right)}^{-\alpha }}{\theta P^{V}{\left(d_{k}^{VM}\right)}^{-\alpha }+P^{M}{\left(d_{k}^{M}\right)}^{-\alpha }}. \end{array}$$

Substitute Equation (1) into $\rho_{nk}^{2}$. Because g∼exp(1) and let $t_{1}=\frac{\beta }{P^{M}{\left(d^{MV}\right)}^{-\alpha }}$, we can derive the following equation:

(11) $$\begin{array}{cc} \rho_{nk}^{2}= & \;P\left(\frac{P^{M}g_{k}^{MV}{\left(d^{MV}\right)}^{-\alpha }}{I_{nk}^{VV}}\leq \beta \right)\\ = & \;P\left(g_{k}^{MV}\leq \frac{\beta I_{nk}^{VV}}{P^{M}{\left(d^{MV}\right)}^{-\alpha }}\right)\\ = & \;1-E\left(\exp\left(-I_{nk}^{VV}t_{1}\right)\right). \end{array}$$

Because the platoons are distributed as a 1D PPP, we can derive the following equation, referring to the result in Reference [32]:

(12) $$E\left(\exp\left(-I_{nk}^{VV}t_{1}\right)\right)=\exp\left(-\frac{2\pi \lambda {\left(t_{1}P^{V}\right)}^{\frac{1}{\alpha }}}{\alpha \sin\left(\frac{\pi }{\alpha }\right)}\right).$$

We can replace $t_{1}$ with $\frac{\beta }{P^{M}{\left(d_{k}^{MV}\right)}^{-\alpha }}$ in reverse in Equation (12) and substitute Equation (12) into Equation (11); then, $\rho_{nk}^{2}$ can be derived as follows:

(13) $$\rho_{nk}^{2}=1-\exp\left(-\frac{2\pi \lambda {\left(\frac{\beta P^{V}}{P^{M}}\right)}^{\frac{1}{\alpha }}d^{MV}}{\alpha \sin\left(\frac{\pi }{\alpha }\right)}\right).$$

Let $\delta_{nk}\left(t\right)$ denote the binary variable that indicates whether channel *k* is available to member *n* in the platoon in slot *t*. By substituting Equations (10) and (13) into Equation (8), we can derive the probability that channel *k* is available to member *n* in the platoon as follows:

(14) $$\begin{array}{cc} \rho_{nk}= & \;P\left(\delta_{nk}\left(t\right)=1\right)\\ = & \;1-b_{k}+b_{k}\left(\frac{P^{M}{\left(d_{k}^{M}\right)}^{-\alpha }}{\theta P^{V}{\left(d_{k}^{VM}\right)}^{-\alpha }+P^{M}{\left(d_{k}^{M}\right)}^{-\alpha }}\right)\left(1-\exp\left(-\frac{2\pi \lambda {\left(\frac{\beta P^{V}}{P^{M}}\right)}^{\frac{1}{\alpha }}d^{MV}}{\alpha \sin\left(\frac{\pi }{\alpha }\right)}\right)\right). \end{array}$$

### 2.4. Non-Outage Probability Calculated by Stochastic Geometry

The non-outage probability is used to represent the quality of the channel [22,23]; the non-outage event is defined as a successful packet transmission on the condition that the SIR is larger than the threshold θ:

(15) $$\begin{array}{cc} P\left(SIR_{nk}^{V}\geq \theta \right)= & \;P\;\left(g_{nk}^{V}\geq \frac{I_{nk}^{V}\theta }{P^{V}{\left(d^{V}\right)}^{-\alpha }}\right)\\ = & \;E\left(\exp\left(\frac{-I_{nk}^{V}\theta }{P^{V}{\left(d^{V}\right)}^{-\alpha }}\right)\right). \end{array}$$

Let symbol $t_{2}=\frac{\theta }{P^{V}{\left(d^{V}\right)}^{-\alpha }}$. Then, by substituting Equation (3) into Equation (15), we can rearrange Equation (15) and obtain:

(16) $$\begin{array}{cc} E\left(\exp\left(\frac{-I_{nk}^{V}\theta }{P^{V}{\left(d^{V}\right)}^{-\alpha }}\right)\right)= & \;E\left(\exp\left(-I_{nk}^{V}t_{2}\right)\right)\\ = & \;E\left(\exp\left(-\left(I_{nk}^{MV}+I_{nk}^{VV}\right)t_{2}\right)\right)\\ = & \;E\left(\exp\left(-I_{nk}^{MV}t_{2}\right)\right)E\left(\exp\left(-I_{nk}^{VV}t_{2}\right)\right). \end{array}$$

The last equality of Equation (16) is derived from the independence between the distribution of the platoons and the location of the MBS. As shown in Equation (12), the latter part of Equation (16) can be derived as follows [33]:

(17) $$E\left(\exp\left(-I_{nk}^{VV}t_{2}\right)\right)=\exp\left(-\frac{2\pi \lambda {\left(t_{2}P^{V}\right)}^{\frac{1}{\alpha }}}{\alpha \sin\left(\frac{\pi }{\alpha }\right)}\right).$$

By substituting Equation (1) into $I_{nk}^{MV}$, because the geographical location and channel occupation of the MUs are broadcast to each platoon leader and g∼exp(1), we can derive the following equation:

(18) $$\begin{array}{cc} E\left(\exp\left(-I_{nk}^{MV}t_{2}\right)\right)= & \;\exp\left(E\left(-t_{2}b_{k}P^{M}g_{k}^{MV}{\left(d^{MV}\right)}^{-\alpha }\right)\right)\\ = & \;\exp\left(-t_{2}b_{k}P^{M}{\left(d^{MV}\right)}^{-\alpha }\right). \end{array}$$

Let $\xi_{nk}\left(t\right)$ denote the binary variable that indicates whether channel *k* is in a non-outage state to member *n* in the platoon in slot *t*. We can replace $t_{2}$ with $\frac{\theta }{P^{V}{\left(d^{V}\right)}^{-\alpha }}$ in reverse in Equations (17) and (18), and substitute Equations (17) and (18) into Equation (16); then, the non-outage probability of a VU is derived as follows:

(19) $$\begin{array}{cc} \varphi_{nk}= & \;P\left(\xi_{nk}\left(t\right)=1\right)\\ = & \;P\left(SIR_{nk}^{V}\geq \theta \right)\\ = & \;\exp\left(-\frac{2\pi \lambda d^{V}\theta^{\frac{1}{\alpha }}}{\alpha \sin\left(\frac{\pi }{\alpha }\right)}\right)\exp\left(-b_{k}\frac{P^{M}\theta }{P^{V}}{\left(\frac{d^{MV}}{d^{V}}\right)}^{-\alpha }\right). \end{array}$$

### 2.5. Data Queue Model

To represent the dynamic of the backlogged data in each VU, we define the data queue, denoted as $Q_{n}\left(t\right)$, for member *n* in the platoon in time slot *t*. The service rate is defined as the number of served data packets at time slot *t*, which is denoted as $\mu_{n}\left(t\right)$. The arrival rate is defined as the number of arrived data packets at time slot *t*, which is denoted as $a_{n}\left(t\right)$. Then, the length of queue $Q_{n}\left(t\right)$ is updated as shown in the following equation:

(20) $$Q_{n}\left(t+1\right)=\max\left[Q_{n}\left(t\right)-\mu_{n}\left(t\right),\;0\right]+a_{n}\left(t\right).$$

In this paper, it is assumed that the data arrivals of VUs are mutually independent and the data arrival of member *n* in the platoon is a Poisson process with a parameter that equals $a_{n}$.

At the beginning of each time slot, each platoon leader makes a decision regarding the channel allocation to its members. Let $x_{nk}$ denote the variable of the channel allocation matrix X, which is defined as:

(21) $$x_{nk}\left(t\right)=\left\{\begin{array}{c} 1,\;\mathrm{if}\;\mathrm{channel}\;k\;\mathrm{is}\;\mathrm{allocated}\;\mathrm{to}\;\mathrm{member}\;n\;\mathrm{at}\;\mathrm{time}\;\mathrm{slot}\;t,\\ 0,\;\mathrm{otherwise}. \end{array}\right.$$

In each slot *t*, the state of each member in the platoon is defined as active or inactive, depending on whether a channel has been allocated to the member. If the member is active, $x_{nk}\left(t\right)=1,\;\exists k\in K$ and if the member is inactive, $x_{nk}\left(t\right)=0,\;\forall k\in K$. For member *n* in the platoon, depending on the relationship between the channel allocation in slot t−1, the decision space in slot *t* can be classified into five options, represented by $S=\left\{D_{1},\;D_{2},\;D_{3},\;D_{4},\;D_{5}\right\}$:

(1) $D_{1}$: $x_{nk}\left(t-1\right)=1,\;x_{nk}\left(t\right)=1,\;\exists k\in K$, use the same channel as in the slot t−1;

(2) $D_{2}$: $x_{nk}\left(t-1\right)=1,x_{nk^{\prime }}\left(t-1\right)=0,\;\;x_{nk}\left(t\right)=0,\;x_{nk^{\prime }}\left(t\right)=1,\;k\neq k^{\prime }$, switch to another channel;

(3) $D_{3}$: $x_{nk}\left(t-1\right)=1,\;x_{nk^{\prime }}\left(t\right)=0,\;\forall k^{\prime }\in K$, change to an inactive state;

(4) $D_{4}$: $x_{nk}\left(t-1\right)=0,\;\forall k\in K,\;x_{nk^{\prime }}\left(t\right)=1$, change to an active state;

(5) $D_{5}$: $x_{nk}\left(t-1\right)=0,\;x_{nk^{\prime }}\left(t\right)=0,\;\forall k,\;k^{\prime }\in K$, maintain the inactive state.

The service rate $\mu_{n}\left(t\right)$ of member *n* in slot *t* is dependent on the channel allocation, the availability variable $\delta_{nk}\left(t\right)$, and the non-outage variable $\xi_{nk}\left(t\right)$, which can be derived as follows:

(22) $$\mu_{n}\left(t\right)=\left\{\begin{array}{cc} \sum_{k\in K}x_{nk}\left(t-1\right)\delta_{nk}\left(t\right)\xi_{nk}\left(t\right), & \;\mathrm{if}\;D\left(t\right)=D_{1},\\ \sum_{k\in K}x_{nk}\left(t\right)\delta_{nk}\left(t\right)\xi_{nk}\left(t\right), & \;\mathrm{if}\;D\left(t\right)=D_{2},\\ 0, & \;\mathrm{if}\;D\left(t\right)=D_{3},\\ \sum_{k\in K}x_{nk}\left(t\right)\delta_{nk}\left(t\right)\xi_{nk}\left(t\right), & \;\mathrm{if}\;D\left(t\right)=D_{4},\\ 0, & \;\mathrm{if}\;D\left(t\right)=D_{5}. \end{array}\right.$$

As represented in Equations (14) and (19), the availability probability and the non-outage probability depend on the locations of the MBS, the platoons, and the MUs. Because the network is dynamic, the channel allocation has to be updated. We define the re-allocation as the allocated channel that differs from that in the prior slot; this includes the decisions $D_{2}$ and $D_{4}$. Let r(t) denote the indicator of re-allocation of a platoon, which is defined as follows:

(23) $$r\left(t\right)=\left\{\begin{array}{cc} 1, & \;\mathrm{if}\;D\left(t\right)=D_{2},\;\exists n\in N,\\ 1, & \;\mathrm{if}\;D\left(t\right)=D_{4},\;\exists n\in N,\\ 0, & \;\mathrm{otherwise}. \end{array}\right.$$

The resource re-allocation results in additional signal overhead and latency [22,23]. First, if the communication is active in the prior slot and then switches to another channel, the current transmission needs to be terminated. Second, synchronization between the transmitter and receiver is required if there is re-allocation. To decrease the additional signal overhead and latency in the network, the execution of re-allocation is considered a cost. Therefore, the rate of re-allocation is limited and is below a certain threshold, denoted as γ, in our channel allocation optimization problem.

### 2.6. Dynamic Maximization Problem of the Number of Service-Guaranteed Users

A VU is regarded as a service-guaranteed user if the sum of the service rates is always larger than that of the data arrival rates in a window denoted as *W*. Let $z_{n}\left(t\right)$ be the notation of user *n* to be service-guaranteed in slot *t*, which is defined as follows:

(24) $$z_{n}\left(t\right)=\left\{\begin{array}{c} z_{n}\left(t-1\right),\;\mathrm{if}\;\sum_{\tau =t-W}^{t}\mu_{n}\left(\tau \right)\geq \sum_{\tau =t-W}^{t}a_{n}\left(\tau \right),\;\forall n\in N,\\ 0,\;\mathrm{otherwise}, \end{array}\right.$$

where it is assumed that $z_{n}\left(0\right)=1,\;\forall n\in N$.

Constrained by the limited resources, not all VUs can be allocated to a resource without suffering interference. To guarantee the quality of service, the set of served services at which the scale of demand exceeds the capability of the network has to be determined. To increase the number of service-guaranteed users, the dynamic channel allocation problem can be formulated as follows:

(25) $$\begin{array}{ccc} & \max_{X}\;\;\;f\left(t\right)=\sum_{n=1}^{N}z_{n}\left(t\right) & (25a),\\ s.t. & \sum_{n\in N}x_{nk}\left(t\right)\leq 1,\;\forall \;k\in K & (25b),\\ & \lim_{T\to \infty }\frac{1}{T}\sum_{t=1}^{T}r(t)\leq \gamma & (25c),\\ & x_{nk}\left(t\right)=x_{nk}\left(t-\left(1-r(t)\right)\right),\;\forall \;n\in N,\;k\in K & (25d),\\ & x_{nk}\left(t\right)=\left\{0,1\right\},\;\forall n\in N,\;k\in K & (25e). \end{array}$$

The optimization problem aims to maximize the number of service-guaranteed users. Constraint 1 guarantees the interference mitigation among the VUs in a platoon, where the channel allocation is orthogonal for each member. Constraint 2 is the constraint of the rate of reallocation. Constraint 3 means that the channel allocation remains the same if there is no re-allocation. Constraint 4 ensures that all the elements in the optimization problem are 0/1 variables.

## 3. Dynamic Algorithm of Resource Re-allocation

Due to the highly dynamic environment of vehicular networks, a computationally efficient online resource allocation scheme has to be designed. A stochastic optimization problem can be transformed into a series of deterministic optimization problems using the Lyapunov optimization and can then be used to decrease the computational complexity for the resource allocation [32]. Therefore, the problem in Equation (25) is solved by the Lyapunov optimization.

The time-averaged expected service rate of member *n* in the platoon is denoted as $\bar{\mu_{n}}$ , which satisfies:

(26) $$\lim_{T\to \infty }\frac{1}{T}\sum_{t=1}^{T}E\left\{\mu_{n}\left(t\right)\right\}=\bar{\mu_{n}}.$$

Based on the stability conditions of the queue, as stated in [32], the sufficient condition for stability occurs if $a_{n}\leq \bar{\mu_{n}}$.

### 3.1. Virtual Queue

Constraint 2 in *P* is the constraint of the rate of re-allocation, where re-allocation is the cost of the optimization. To transform Constraint 2 with time-averaging into the queueing stability problem, the notion of a virtual cost queue is employed [32]. The virtual cost queue for the re-allocation rate of the platoon is denoted as R(t) and the corresponding queue update equation is:

(27) $$R\left(t+1\right)=\max\left[R(t)-\gamma ,\;0\right]+r\left(t\right).$$

The variable R(t) represents the backlogs of the virtual queue with a constant service rate γ and an input process r(t). If the stability of the queue can be guaranteed, the time-average of the arrival rate r(t) is no more than the constant rate γ. That is, Constraint 2 in *P* can be guaranteed.

### 3.2. Lyapunov Optimization

In this study, the Lyapunov function, denoted as L(t), is defined as follows:

(28) $$L\left(t\right)=\sum_{n=1}^{N}{\left(z_{n}\left(t\right)Q_{n}\left(t\right)\right)}^{2}+{\left(R(t)\right)}^{2}.$$

The Lyapunov drift is $\Delta L\left(t\right)=L\left(t+1\right)-L\left(t\right)$, which represents the trend of the change in the data queues of the service-guaranteed user $Q^{n}\left(t\right)$ and the virtual cost queue R(t). To guarantee the stability of the data queues and the virtual cost queue, the drift has to be decreased. That means that the platoon leader needs to decrease the number of active users to increase the service rate of the active users; the backlogs of the active users are decreased and there is no need for frequent re-allocation among the users. However, this is contrary to the objective of the optimization problem. Therefore, to stabilize the queues and optimize the objective of *P* simultaneously, the drift-plus-penalty function is defined as follows:

MinΔL(t)−Vf(t),

where *V* is a control parameter that determines the trade-off between the drift of the queues and the objective of *P* in Equation (25).

As in the lemma presented in Reference [32], if there are four nonnegative real members–*A*, *B*, ω,η, and $A\leq \max\left[B-\omega ,\;0\right]+\eta$–then $A^{2}\leq B^{2}+\omega^{2}+\eta^{2}-2B\left(\omega -\eta \right)$. Thus, the drift-plus-penalty function can be derived as:

(29) $$\begin{array}{ccc} \Delta L(t)-Vf(t) & = & L\left(t+1\right)-L\left(t\right)-Vf\left(t\right)\\ & = & \;\sum_{n=1}^{N}{\left(z_{n}\left(t+1\right)Q_{n}\left(t+1\right)\right)}^{2}+{\left(R\left(t+1\right)\right)}^{2}-\sum_{n=1}^{N}{\left(z_{n}\left(t\right)Q_{n}\left(t\right)\right)}^{2}-{\left(R(t)\right)}^{2}\\ & & \;-V\sum_{n=1}^{N}z_{n}\left(t\right)\\ & \leq & \sum_{n=1}^{N}z_{n}\left(t\right)\left({\left(\mu_{n}\left(t\right)\right)}^{2}+{\left(a_{n}\left(t\right)\right)}^{2}-2Q_{n}\left(t\right)\left(\mu_{n}\left(t\right)-a_{n}\left(t\right)\right)\right)+\gamma^{2}\\ & & \;+{\left(r(t)\right)}^{2}-2R\left(t\right)\left(\gamma -r(t)\right)-V\sum_{n=1}^{N}z_{n}\left(t\right). \end{array}$$

Therefore, to minimize the upper bound of the drift-plus-penalty function in Equation (29), the channel allocation scheme is proposed as follows:

(30) $$\max_{X}\;\;\sum_{n=1}^{N}z_{n}\left(t\right)\left(2Q_{n}\left(t\right)\left(\mu_{n}\left(t\right)-a_{n}\left(t\right)\right)+V\right)-2R\left(t\right)r\left(t\right)$$

under the constraints (25b)∼(25e).

By minimizing Equation (30), we obtain the following:

(31) $$\begin{array}{cc} E\left\{\Delta L(t)-Vf(t)\right\} & \leq \Theta +E\left\{\sum_{n=1}^{N}z_{n}^{*}\left(t\right)\left(-2Q_{n}\left(t\right)\left(\mu_{n}^{*}\left(t\right)-a_{n}\left(t\right)\right)\right)\right\}\\ & \;-2E\left\{R\left(t\right)\left(\gamma -r^{*}\left(t\right)\right)\right\}-VE\left\{f^{*}\left(t\right)\right\}, \end{array}$$

where Θ is a constant that satisfies the following inequation:

$\Theta \geq E\left\{\sum_{n=1}^{N}\left({\left(\mu_{n}\left(t\right)\right)}^{2}+{\left(a_{n}\left(t\right)\right)}^{2}\right)+{\left(r(t)\right)}^{2}+\gamma^{2}\right\},\;f^{*}\left(t\right),$ and $r^{*}\left(t\right)$ are the results of all possible policies (including the optimal one).

Furthermore, we use the time-average of Equation (31) and rearrange the equation to obtain:

(32) $$\begin{array}{cc} & \;\lim_{T\to \infty }\frac{1}{T}\sum_{t=1}^{T}E\left\{\sum_{n=1}^{N}z_{n}^{*}\left(t\right)\left(2Q_{n}\left(t\right)\left(\mu_{n}^{*}\left(t\right)-a_{n}\left(t\right)\right)\right)-Vf\left(t\right)\right\}\\ \leq & \;\Theta +\lim_{T\to \infty }\frac{1}{T}E\left\{L(t)-L(1)\right\}-2\lim_{T\to \infty }\frac{1}{T}\sum_{t=1}^{T}E\left\{R\left(t\right)\left(\gamma -r^{*}\left(t\right)\right)\right\}-V\lim_{T\to \infty }\frac{1}{T}\sum_{t=1}^{T}E\left\{f^{*}\left(t\right)\right\}, \end{array}$$

where ε is a small constant such that $\bar{a_{n}\left(t\right)}+\varepsilon \leq \bar{\mu_{n}\left(t\right)}$ for each VU that $z_{n}\left(t\right)=1$. Due to $\lim_{T\to \infty }\frac{1}{T}E\left\{L(t)-L(1)\right\}=0$ and $\lim_{T\to \infty }\frac{1}{T}\sum_{t=1}^{T}E\left\{R\left(t\right)\left(\gamma -r^{*}\left(t\right)\right)\right\}\geq 0$, the lower bound of the expectations of the number of service-guaranteed users can be guaranteed as follows:

(33) $$\begin{array}{cc} \lim_{T\to \infty }\frac{1}{T}\sum_{t=1}^{T}E\left\{2\varepsilon \sum_{n=1}^{N}z_{n}^{*}\left(t\right)Q_{n}^{*}\left(t\right)-Vf\left(t\right)\right\}\leq & \;\Theta -V\lim_{T\to \infty }\frac{1}{T}\sum_{t=1}^{T}E\left\{f^{*}\left(t\right)\right\}\\ VE\left\{\bar{f(t)}\right\}\geq & \;VE\left\{\bar{f^{*}\left(t\right)}\right\}-\Theta +2\varepsilon \lim_{T\to \infty }\frac{1}{T}\sum_{t=1}^{T}\sum_{n=1}^{N}E\left\{z_{n}^{*}\left(t\right)Q_{n}^{*}\left(t\right)\right\}\\ E\left\{\bar{f(t)}\right\}\geq & \;E\left\{\bar{f^{*}\left(t\right)}\right\}+\frac{-\Theta +2\varepsilon \sum_{n\in Z^{*}}\bar{Q_{n}^{*}\left(t\right)}}{V}, \end{array}$$

where $z_{n}^{*}\left(t\right)$ and $Q_{n}^{*}\left(t\right)$ are the set of the service-guaranteed users and the queue of user *n* corresponding to $f^{*}\left(t\right)$ and $r^{*}\left(t\right)$:

(34) $$\sum_{n\in Z}\bar{Q(t)}\leq \sum_{n\in Z^{*}}\bar{Q_{n}^{*}\left(t\right)}\leq \frac{\Theta +V\left(E\left\{\bar{f\left(t\right)-f^{*}\left(t\right)}\right\}\right)}{2\varepsilon }.$$

From Equations (33) and (34), it is found that the constant *V* determines the lower bound of the number of service-guaranteed users and the upper bound of the length of the queue.

### 3.3. Implementation of the Proposed Resource Allocation Scheme and Its Overhead

In summary, the implementation of the proposed resource allocation scheme can be described as follows. First, at the beginning of each time slot, the MBS broadcasts information on the geographical location and channel occupation of all MUs to each platoon leader, and the members send the interference strength, the signal strength, the length of queue, and the data arrival rate to the corresponding platoon leader. Second, each platoon leader calculates $\delta_{nk}\left(t\right)$ and $\xi_{nk}\left(t\right)$ for each member. Lastly, according to the information including $z_{n}\left(t-1\right)$, $Q_{n}\left(t-1\right)$, $a_{n}\left(t-1\right)$, $R\left(t-1\right)$, $\delta_{nk}\left(t\right)$, and $\xi_{nk}\left(t\right)$, each platoon leader determines X by solving the optimization problem in Equation (30) and allocates the channel to members. Therefore, the signaling overhead in the proposed scheme consists of two parts. The first part is the information of MUs transmitted by the MBS to the platoon leader, which includes the geographical location and channel occupation. The second part is the information of VUs transmitted by members to the platoon leader, which includes the interference and the signal strength, the length of queue, and the data arrival rate.

A summary of notations in this paper is presented in Table 1.

## 4. Simulation

In this section, we verify our analytical results and evaluate the proposed scheme using MATLAB (R2017a, The MathWorks, Natick, MA, USA) as the simulation platform. The parameters are listed in Table 2. In this simulation, the MBS is located in the middle of the road and the MUs are uniformly and randomly distributed on the road. To represent the difference of the data arrival rates of multiple users, the data arrival rates of users are uniformly and randomly distributed throughout the segment $\left[a^{\min},\;a^{\max}\right]$.

The parameters including *N*, θ, α, $P^{M}$, $P^{V}$, γ, and Δt refer to those in References [22,23].

### 4.1. Comparison between the Theoretical Calculations and the Simulation Results

In this section, we compare the theoretical calculations with the simulation results of the average availability and the non-outage probabilities.

**Figure 2:**
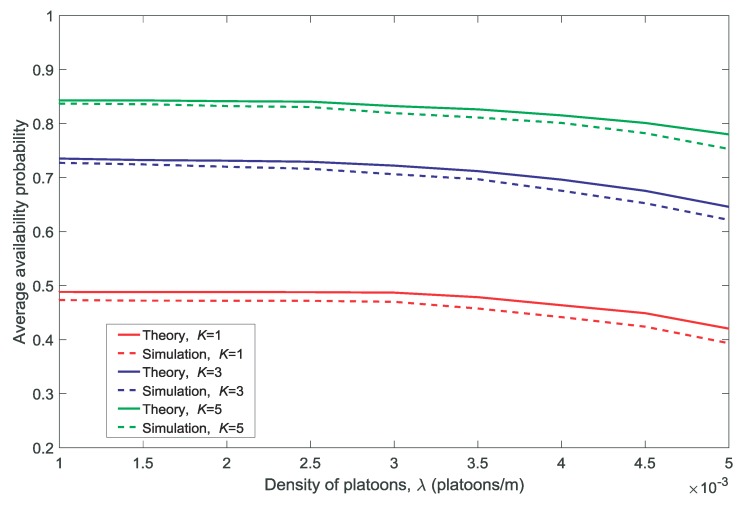
Average availability probability for different densities of platoons $\lambda (d^{V}=10,V=10,N=5)$.

**Figure 3:**
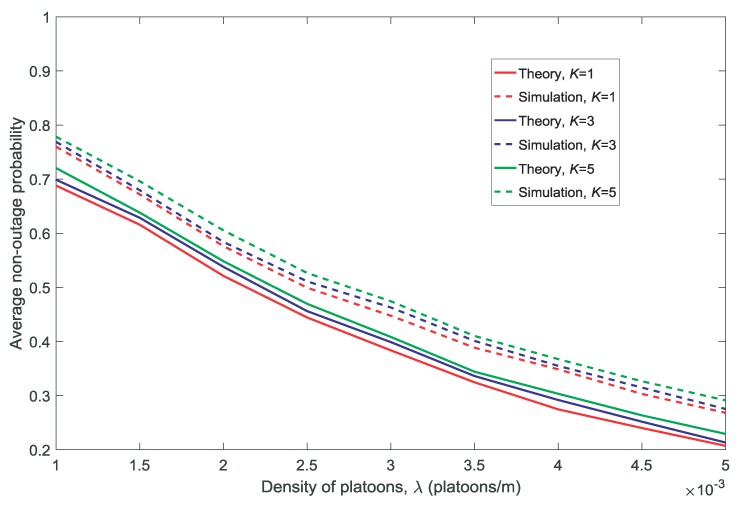
Average non-outage probability for different densities of platoons $\lambda (d^{V}=10,V=10,N=5)$.

**Figure 4:**
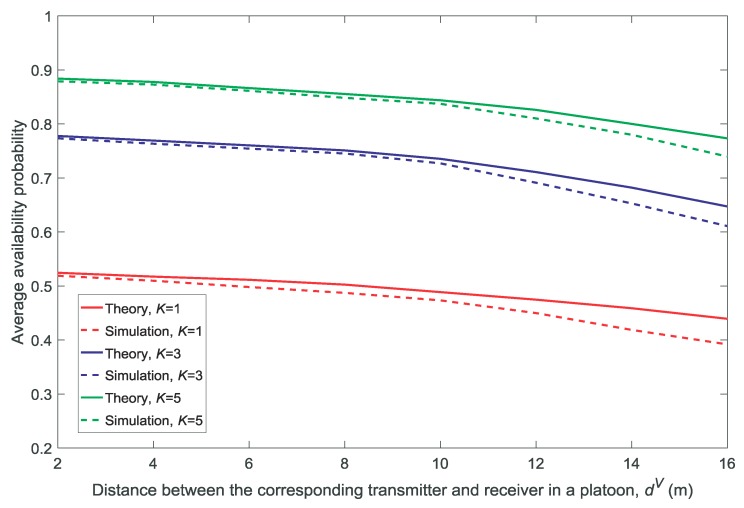
Average availability probability for different distances between the corresponding transmitter and receiver in a platoon $d^{V}\left(\lambda =1/1000,V=10,N=5\right)$.

**Figure 5:**
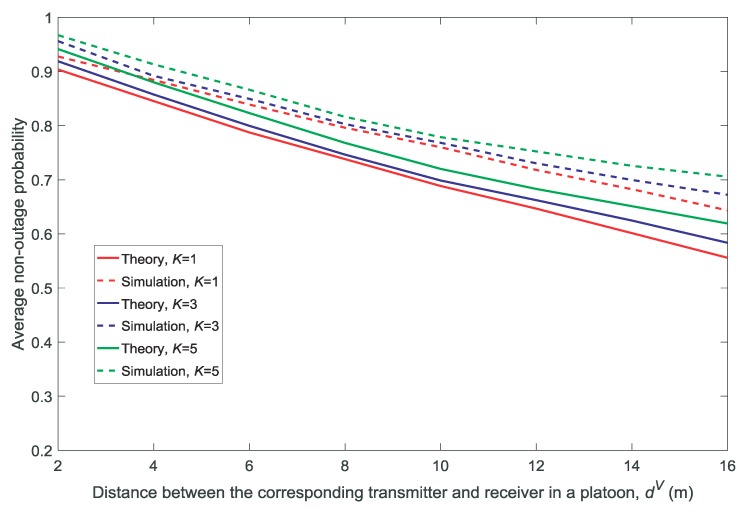
Average non-outage probability for different distances between the corresponding transmitter and receiver in a platoon $d^{V}\left(\lambda =1/1000,V=10,N=5\right)$.

Figure 2 and Figure 3 show the effect of the density of the platoons λ on the average availability probability and average non-outage probability, respectively. Figure 4 and Figure 5 show the effect of the distance between the corresponding transmitter and receiver in a platoon $d^{V}$ on the average availability probability and average non-outage probability, respectively. As shown in Figure 2, Figure 3, Figure 4 and Figure 5, the theoretical values of the average availability probability are slightly higher than those in the simulation, whereas the opposite can be observed for the average non-outage probability. This is because all the channels are assumed to be occupied by each platoon in the theoretical calculations. However, if a channel is unavailable for all members according to Conditions 1 and 2, the platoon leader will not allocate the channel to its members. That is to say, the interference from the vehicles is higher in theory than in reality. Therefore, the estimate of the availability probability is too high because Condition 2 is relaxed in the theoretical calculation, whereas the estimate of the average non-outage probability is too low due to the excessive interference in the theoretical calculation.

It is observed in Figure 2 and Figure 4 that the average availability probability is influenced primarily by the number of channels *K* because the interference between the VU and MU decreases with increasing *K*. The results in Figure 3 and Figure 5 indicate that the average non-outage probability is influenced primarily by λ and $d^{V}$ because the interference in the network increases with the increase in λ and the desired signal declines with the increase in $d^{V}$.

In addition, Figure 4 and Figure 5 show that the gap between the theoretical values and the simulation results increases with the increase in $d^{V}$. This is because each platoon is regarded as a point in the theoretical calculations by the other platoons and the MU, and the accuracy of the approximation decreases with the increase in $d^{V}$.

### 4.2. The Performance of the Proposed Scheme

In the simulation, we compare the proposed scheme with the resource re-allocation scheme presented in Reference [23] (referred to as Scheme 1 for simplification).

**Figure 6:**
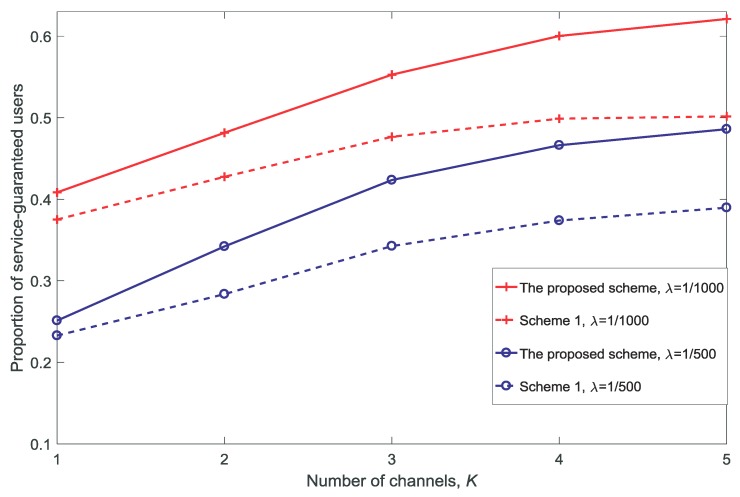
Proportion of service-guaranteed users for different numbers of channels $K(d^{V}\;=\;10,V\;=\;10,N=5)$.

**Figure 7:**
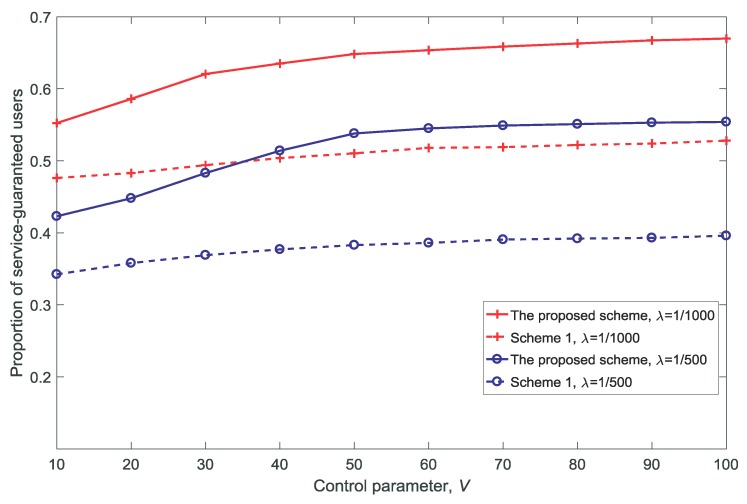
Proportion of service-guaranteed users for different control parameter values $V(d^{V}\;=\;10,K\;=\;3,N\;=\;5)$.

**Figure 8:**
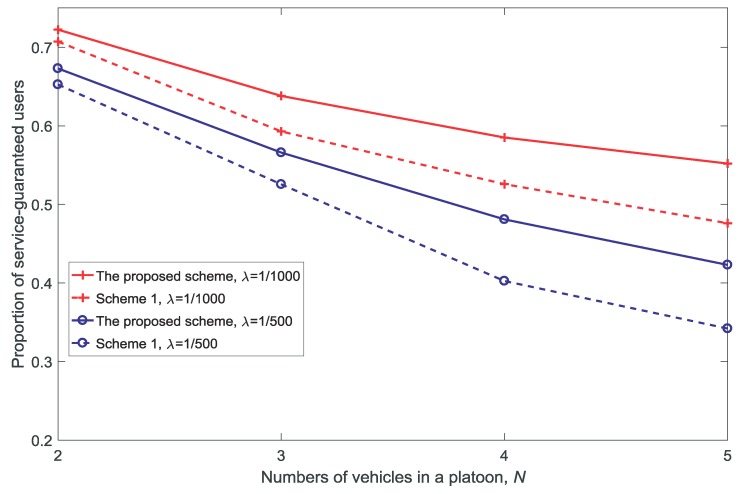
Proportion of service-guaranteed users for different numbers of vehicles in a platoon $N(d^{V}=10,K=3,V=10)$.

**Figure 9:**
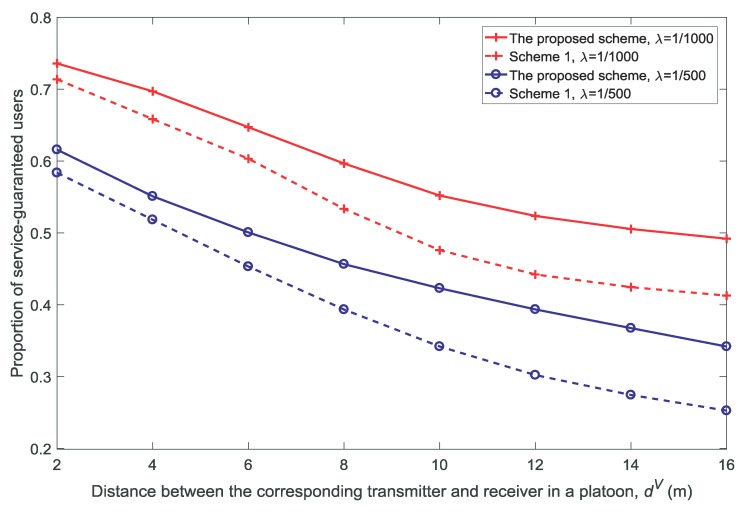
Proportion of service-guaranteed users for different distances between the corresponding transmitter and receiver in a platoon $d^{V}\left(N=5,K=3,V=10\right)$.

Figure 6, Figure 7, Figure 8 and Figure 9 show the proportion of service-guaranteed users for different values of *K*, *V*, *N*, and $d^{V}$, respectively. From Figure 6, Figure 7, Figure 8 and Figure 9, it is observed that the proposed scheme results in a higher proportion of service-guaranteed users than Scheme 1. This is because the proposed scheme selects some of the users to receive guaranteed service by dropping others when the scale of demand exceeds the service capability of the resources, whereas Scheme 1 provides insufficient services to all users. The proportion of service-guaranteed users is higher at λ=1/1000 than that achieved using λ=1/500 because the network is denser at a higher value of λ. In addition, Figure 7 shows that the proportion of service-guaranteed users increases with the increase in *V*. This is because the control parameter is the coefficient of the number of service-guaranteed users in the drift-plus-penalty function. A higher value of *V* indicates a greater focus on the number of service-guaranteed users in the optimization problem, as shown in Equation (30). Figure 8 and Figure 9 show that the proportion of service-guaranteed users decreases with the increase of *N* and $d^{V}$. This is because the network becomes denser with the increase in *N* and the desired signal declines with the increase in $d^{V}$.

**Figure 10:**
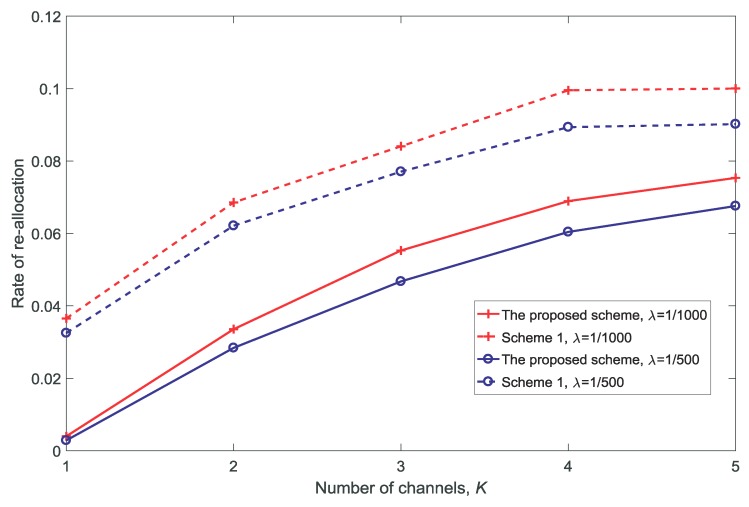
Rate of re-allocation for different numbers of channels $K(d^{V}=10,V\;=\;10,N=5)$.

**Figure 11:**
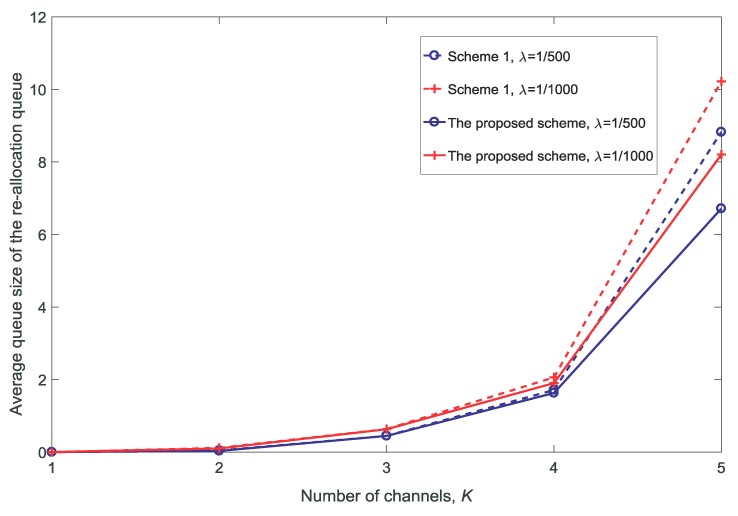
Average queue size of the re-allocation for different numbers of channels $K(d^{V}\;=\;10,V\;=\;10,N=5)$.

Figure 10 and Figure 11 show the rate and the average queue size of re-allocation for different numbers of channels *K*, respectively. It is observed that both the rate and the average queue size of re-allocation increase with the increase in *K*. There are two reasons for this: first, there are more opportunities for the MUs to obtain a channel with better conditions as *K* increases; second, there are more opportunities for the VUs to be guaranteed due to the re-allocation of the channels as *K* increases.

**Figure 12:**
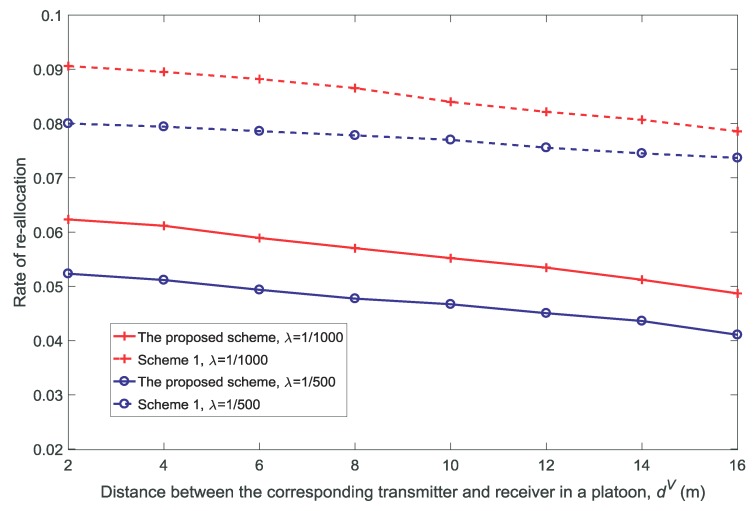
Rate of re-allocation for different distances between the corresponding transmitter and receiver in a platoon $d^{V}\left(K=3,V=10,N=5\right)$.

**Figure 13:**
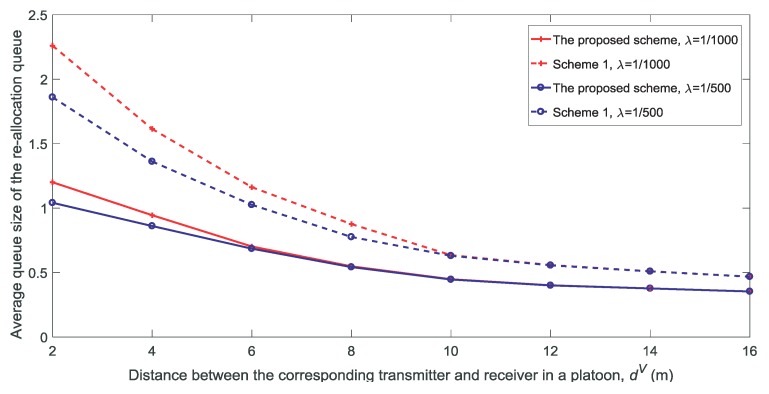
Average queue size of the re-allocation for different distances between the corresponding transmitter and receiver in a platoon $d^{V}\left(K=3,V=10,N=5\right)$.

Figure 12 and Figure 13 show the rate and the average queue size of re-allocation for different values of $d^{V}$, respectively. It is observed that both the rate and the average queue size of re-allocation decrease with the increase in $d^{V}$. As shown in Figure 4 and Figure 5, as $d^{V}$ increases, the availability probability remains almost constant and the non-outage probability decreases. Therefore, the re-allocation decreases with the decrease in the number of channels that satisfies $\xi_{nk}\left(t\right)=1$.

**Figure 14:**
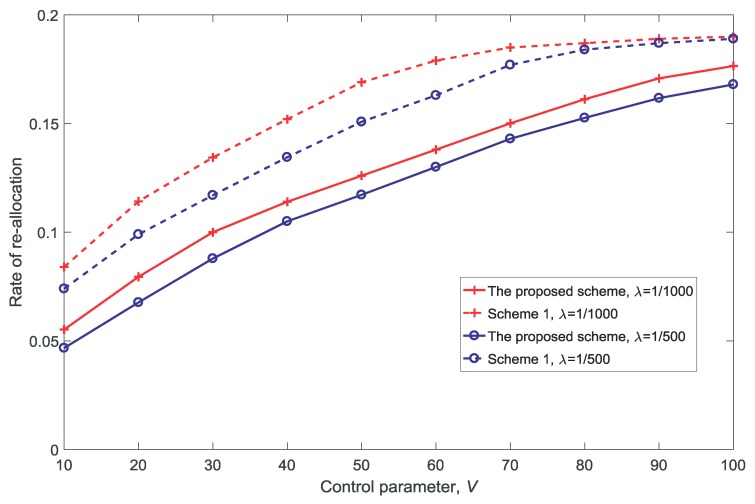
Rate of re-allocation for different control parameter values $V(d^{V}=10,K=3,N=5)$.

Figure 14 shows the rate of re-allocation for different control parameter values *V*. With the increase in *V*, it can be seen that the rate of re-allocation increases. This is because re-allocation is used to increase the number of service-guaranteed users, which can be seen from Figure 7. Figure 7 and Figure 14 show that there is a trade-off between the number of service-guaranteed users and the re-allocation performance; moreover, the results depend on the value of *V*.

**Figure 15:**
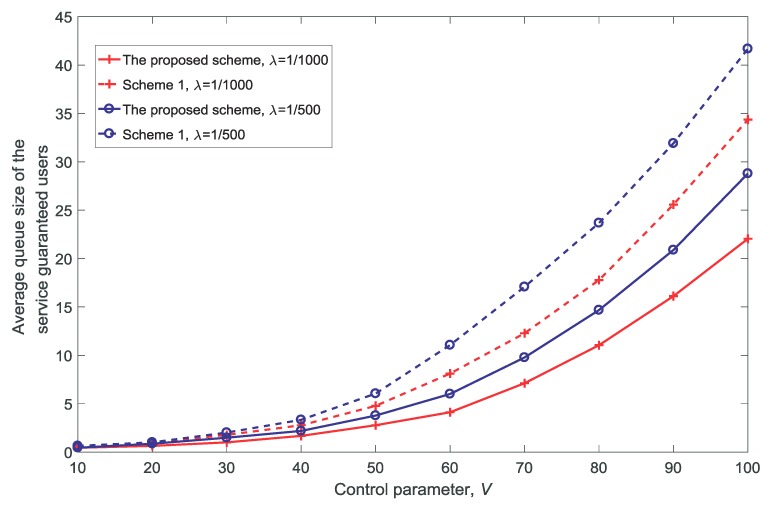
Average queue size of the service-guaranteed users for different control parameter values $V(d^{V}=10,K=3,N=5)$.

Figure 15 shows the average queue size of the service-guaranteed users for different control parameter values *V*. The channels are shared among multiple users and as the number of service-guaranteed users increases, the wait time increases.

**Figure 16:**
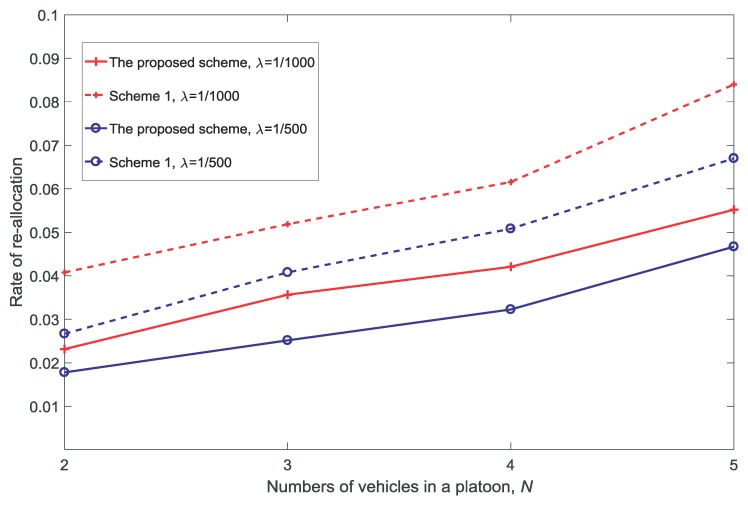
Rate of re-allocation for different numbers of vehicles in a platoon $N(d^{V}\;=\;10,K\;=\;3,V\;=\;10)$.

**Figure 17:**
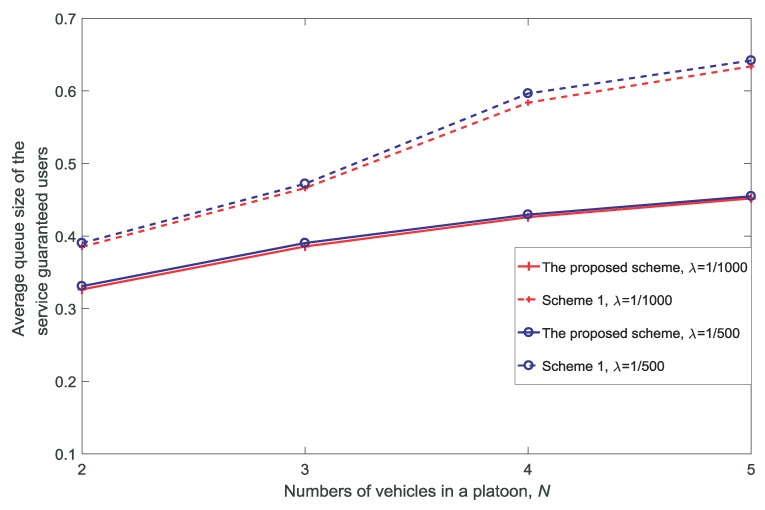
Average queue size of the service-guaranteed users for different numbers of vehicles in a platoon $N(d^{V}=10,K=3,V=10)$.

Figure 16 and Figure 17 show the rate of re-allocation and the average queue size of the service-guaranteed users for different values of *N*. With the increase in *N*, it can be seen that the rate of re-allocation and average queue size of the service-guaranteed users increase. This is because re-allocation is necessary to provide adequate service for the increased number of users, and the service rates of users decrease with the increase in *N* due to the limited resources.

From Figure 10, Figure 12, Figure 14 and Figure 16, it is observed that the proposed scheme results in a lower rate of re-allocation than that achieved using Scheme 1. This is because the proposed scheme selects only some of the users to service, dropping others when the scale of demand exceeds the service capability of the resources. In contrast, Scheme 1 requires more re-allocation among all users as none of users are dropped. In addition, it is observed that the rate of re-allocation is lower at λ=1/500 than at λ=1/1000. As shown in Figure 2 and Figure 3, as λ increases, the availability probability remains almost constant and the non-outage probability decreases. Therefore, the re-allocation at λ=1/500 decreases with the decrease in the number of channels that satisfies $\xi_{nk}\left(t\right)=1$.

## 5. Conclusions

In this paper, we proposed a time dynamic optimization problem that constrains the network re-allocation rate to increase the number of guaranteed services at a low-cost in a resource-limited vehicular network. To decrease the computational complexity, we converted the time dynamic optimization problem to a deterministic optimization problem using the Lyapunov optimization theory. The simulation results demonstrated the validity of the analytical results. Compared with a similar algorithm reported in Reference [23], the proposed scheme provides about 15 percent more service-guaranteed users.

## Figures and Tables

**Table 1:** Summary of notations.

Notations	Meaning
Φ	The set of platoons
λ	The density of platoons
N	The set of vehicles in a platoon
*N*	The cardinality of N
$K=\left\{1,\;2,\;\ldots ,\;K\right\}$	The set of orthogonal channels
$N^{M}$ and $N^{P}$	The number of microcell users (MUs) and the number of platoons
θ	The threshold of the signal-to-interference ratio (SIR)
$P^{M}$ and $P^{V}$	The transmit power of the microcell base station (MBS) and the transmit power of the vehicle user (VU)
α	The factor of the path loss
*n*	The member in the platoon
$I_{nk}^{MV}$	The interference from the MBS to member *n* in the platoon in channel *k*
$g_{k}^{MV}$	The small-scale fading from the MBS to member *n* in the platoon in channel *k*
$d^{MV}$	The distance between the MBS and the platoon
$p^{M}$	The probability of a channel being occupied by an MU
$I_{nk}^{VV}$	The aggregated interference from the other platoons to member *n* in the platoon in channel *k*
$g_{qk}^{VV}$	The small-scale fading from the VU interferer *q* to member *n* in the platoon in channel *k*
$d_{q}^{VV}$	The distance between the VU interferer *q* and the platoon
$I_{nk}^{V}$	The total aggregated interference from the MBS and the other platoons to member *n* in the platoon in channel *k*
$SIR_{nk}^{V}$	The SIR of member *n* in the platoon in channel *k*
$g_{nk}^{V}$	The small-scale fading from the corresponding transmitter to member *n* in the platoon in channel *k*
$d^{V}$	The distance between the corresponding transmitter and receiver in the platoon
$g_{k}^{M}$	The small-scale fading from the MBS to the corresponding MU that is active in channel *k*
$d_{k}^{M}$	The distance between the MBS and the corresponding MU that is active in channel *k*
$g_{k}^{VM}$	The small-scale fading from the VU interferer to the MU that is active in channel *k*
$d_{k}^{VM}$	The distance between the VU interferer and the MU that is active in channel *k*
β	The threshold of the ratio of the interference from the MBS to the interference from the other platoons
$b_{k}$	The binary variable of channel allocation for the MUs in channel *k*
$\rho_{nk}$	The availability probability of channel *k* for member *n* in the platoon
	
$\delta_{nk}\left(t\right)$	The binary variable that indicates whether channel *k* is available to member *n* in the platoon at time slot *t*
$\phi_{nk}$	The non-outage probability of channel *k* for member *n* in the platoon
	
$\xi_{nk}\left(t\right)$	The binary variable that indicates whether channel *k* is in a non-outage state for member *n* in the platoon at time slot *t*
$Q_{n}\left(t\right)$	The data queue for member *n* in the platoon at time slot *t*
$\mu_{n}\left(t\right)$	The rate of service for member *n* in the platoon at time slot *t*
$a_{n}$	The rate of data arrival for member *n* in the platoon
X and $x_{nk}$	The channel allocation matrix and its element
$S=\left\{D_{1},\;D_{2},\;D_{3},\;D_{4},\;D_{5}\right\}$	The set of decisions for member *n* in the platoon
r(t)	The indicator of re-allocation at time slot *t*
γ	The threshold of re-allocation rate
$z_{n}\left(t\right)$	The notation that indicates whether member *n* in the platoon is a service-guaranteed user at time slot *t*
R(t)	The virtual cost queue for the re-allocation rate of the platoon at time slot *t*
L(t) and ΔL(t)	The Lyapunov function and the Lyapunov drift at time slot *t*
*V*	The control parameter

**Table 2:** Simulation parameters.

Simulation Parameters	Value
The threshold of the SIR, θ	5
The path loss factor, α	4
The transmit power of the MBS, $P^{M}$	10 w
The transmit power of the VU, $P^{V}$	3 w
The threshold of the re-allocation rate, γ	0.2
The length of each slot, Δt	10 ms
The velocity of the platoon and MU	40 km/h
The length of the road	2000 m
The maximum data arrival rate of the VUs, $a^{\max}$	1 packet/slot
The minimum data arrival rate of the VUs, $a^{\min}$	0.1 packet/slot
The number of MUs, $N^{M}$	2
The threshold of the ratio of the interference from the MBS to the interference from the other platoons, β	1
The window of the service-guaranteed user, *W*	10

## Abbreviations

The following abbreviations are used in this manuscript:

ITS	intelligent transportation system
V2V	vehicle-to-vehicle
V2I	vehicle-to-infrastructure
C-V2X	cellular vehicle-to-everything
RSU	roadside unit
VU	vehicular user
MU	macrocell user
MBS	macrocell base station
1D PPP	one-dimensional Poisson point process
SIR	signal-to-interference-ratio
